# Upregulation of Phospholipase C Gene Expression Due to Norepinephrine-Induced Hypertrophic Response

**DOI:** 10.3390/cells11162488

**Published:** 2022-08-11

**Authors:** Paramjit S. Tappia, Naranjan S. Dhalla

**Affiliations:** 1 Asper Clinical Research Institute, St. Boniface Hospital, Winnipeg, MB R2H 2A6, Canada; 2 Institute of Cardiovascular Sciences & Department of Physiology & Pathophysiology, Max Rady College of Medicine, Rady Faculty of Health Sciences, University of Manitoba, Winnipeg, MB R3T 2N2, Canada

**Keywords:** phospholipase C isozymes, cardiomyocytes, signal transduction, cardiac gene expression, cardiac cell growth, cardiac hypertrophy

## Abstract

The activation of phospholipase C (PLC) is thought to have a key role in the cardiomyocyte response to several different hypertrophic agents such as norepinephrine, angiotensin II and endothelin-1. PLC activity results in the generation of diacylglycerol and inositol trisphosphate, which are downstream signal transducers for the expression of fetal genes, increased protein synthesis, and subsequent cardiomyocyte growth. In this article, we describe the signal transduction elements that regulate PLC gene expression. The discussion is focused on the norepinephrine- α_1_-adrenoceptor signaling pathway and downstream signaling processes that mediate an upregulation of PLC isozyme gene expression. Evidence is also indicated to demonstrate that PLC activities self-regulate the expression of PLC isozymes with the suggestion that PLC activities may be part of a coordinated signaling process for the perpetuation of cardiac hypertrophy. Accordingly, from the information provided, it is plausible that specific PLC isozymes could be targeted for the mitigation of cardiac hypertrophy.

## 1. Introduction

It is well established that cardiac hypertrophy is associated with concomitant alterations in the expression levels of many genes. In addition, cardiomyocyte growth is due to an increase in protein content in the cardiomyocyte as opposed to an increase in cell numbers because adult cardiomyocytes do not have the capacity to proliferate as they are terminally differentiated [1]. Accordingly, the cardiomyocyte hypertrophic response is characterized by increases in cardiomyocyte size, RNA concentration, and protein synthesis as well as expression of fetal genes including atrial natriuretic factor (ANF) [2,3,4,5,6,7,8,9,10,11,12,13] due to the activation of several transcription factors [14,15]. Furthermore, a transient activation of the c-Fos and c-Jun complex (AP-1) also occurs in the early response to hypertrophic stimuli [14,16]. In fact, cumulative evidence has determined that both AP-1 complex and ANF induce the activation of several hypertrophic genes [14,17,18,19].

The increases in c-Fos mRNA and protein synthesis in response to norepinephrine are attributed to the activation of the α_1_-adrenoceptor (AR) [14,18,20,21]. A transient increase of c-Jun expression has been reported in response to mechanical overload [22]. Cardiomyocyte transfection with a dominant negative c-Jun has been shown to prevent the increases in protein synthesis and atrial natriuretic peptide mRNA in response to the AR agonist, phenylephrine [23]. In contrast, a marked activation of the ANF gene promoter has been observed in cardiomyocytes overexpressing c-Jun [20]. On the other hand, pretreatment of neonatal rat cardiomyocytes, in vitro, with PLC antisense oligonucleotides was demonstrated to prevent the upregulation of c-Fos and c-Jun due to insulin-like growth factor-1 [24]. These authors suggested that signal transduction by specific phospholipase C (PLC) isozymes could have an important role in the regulation of these transcription factors.

**Figure 1:**
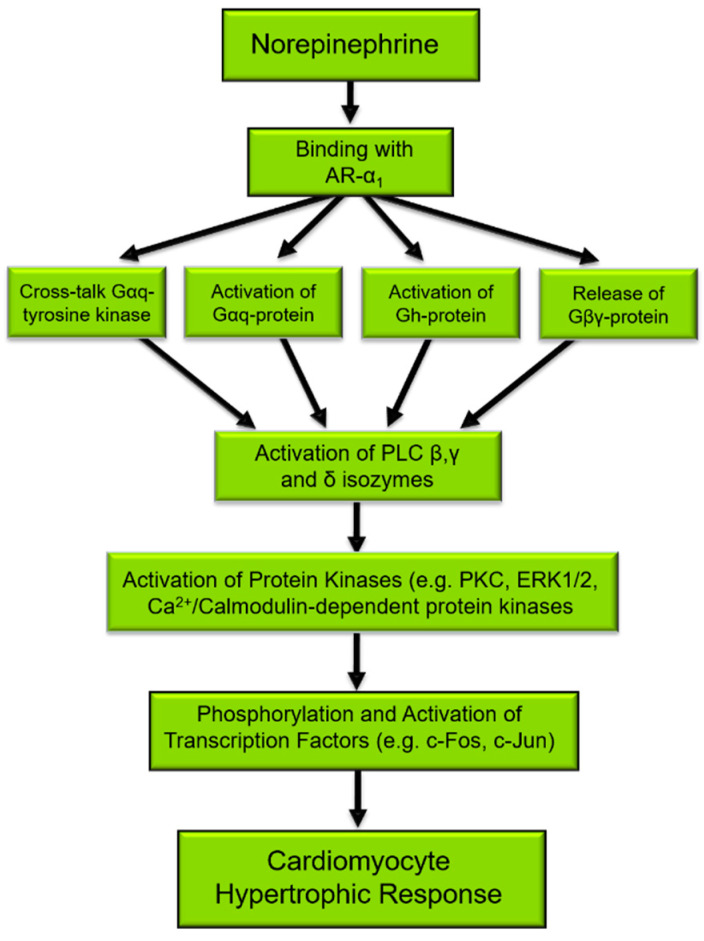
Norepinephrine-induced signal transduction resulting in cardiomyocyte hypertrophy. α_1_-AR, α_1_-adrenoceptors; Gαq, G_q_ protein alpha subunit; PLC, phospholipase C. There are several proteins that are phosphorylated such as ERK1/2, PKC, Ca^2+^/Calmodulin-dependent kinase, and JNK [32], which in turn phosphorylate and activate transcription factors such as c-Fos and c-Jun leading to the gene expression of PLC as well as the expression of fetal genes that are characteristic of cardiac hypertrophy [9,33].

The activation of PLC is considered a primary signaling event in the regulation of diverse cellular functions [25,26]. Several different agents, such as neurohormones and growth factors, induce phenotypic changes in cardiomyocytes that are characteristic of the hypertrophic response and activate PLC [27,28]. Mechanical stretching of isolated cardiomyocytes or in vivo due to pressure/volume overload also results in the activation of the PLC signal transduction pathway [4,29,30,31] as well as induces characteristic features of cardiac hypertrophy. Figure 1 depicts the norepinephrine-mediated signal transduction events that result in cardiomyocyte cell growth, for which PLC is an important and integral component. Indeed, the phosphorylation of target proteins [32], the activation of transcription factors, and subsequent gene expression [9,33] in the cardiomyocyte hypertrophic response to norepinephrine may all be initiated by PLC activation.

Although animal models have facilitated in identifying alterations in gene expression during cardiac hypertrophy, the use of in vitro systems has permitted further delineation of the signal transduction processes and molecular events implicated in increasing cardiomyocyte growth during the response to different hypertrophic stimuli. Thus, in this review, it is intended to describe changes in PLC isozyme gene and protein expression as well as isozyme activities in cardiac hypertrophy due to different etiologies and in isolated cardiomyocytes in response to norepinephrine. In addition, we also discuss the in vivo as well as in vitro signal transduction elements and the identity of the transcription factors that regulate PLC gene expression. Furthermore, the available evidence is presented to show that PLC regulates its own gene expression. Overall, the contention of this article is that PLC might constitute a mechanism for the perpetuation of the hypertrophic process and ultimately its transition to heart failure and may therefore be an important molecular and pharmacological target.

## 2. Regulation of Cardiac PLC Isozymes

There are now 13 families of PLC isozymes that are categorized into 6 classes; PLC β, δ, γ, ε, ζ, and η isozymes [34,35], and are known to be differentially expressed in mammalian cells [36]. Phosphoinositide-specific PLC activity results in the hydrolysis of membrane phosphatidylinositol-4,5-bisphosphate (PIP_2_) to generate inositol-1,4,5-trisphosphate (IP_3_) and 1,2-diacylglycerol (DAG) [37]. Both these lipid molecules activate downstream signal transduction events for cardiac hypertrophy. In this regard, the interaction of IP_3_ with its receptors stimulates the release of Ca^2+^ from intracellular stores, whereas DAG activates protein kinase C (PKC) isoforms. Although there is now a large body of information that has established the involvement of specific PKC isoforms in cardiomyocyte growth [38,39], IP_3_/Ca^2+^ are also considered to be important signaling elements for cardiac hypertrophy [6,9,40,41].

PLC β, δ, γ, and ε [42,43,44,45,46,47] are the major myocardial PLC isozymes and are known to be activated by different mediators including G proteins, tyrosine kinases, and calcium [48]. The PLC β family has four types of isozymes (β_1_, β_2_, β_3_, and β_4_) [36]. PLC β_4_ has been reported to exhibit higher expression level in the heart relative to PLC β_1_, β_2_, and β_3_ [49]. The mechanisms involved in the activation of PLC β_1_ and PLC β_3_ isozymes are the most well studied among PLC isozymes in the heart. In this regard, norepinephrine and other α_1_-AR agonists are known to stimulate PLC β isozyme activities through Gqα [50]. In addition, unlike PLC β_4_, PLC β_1-3_ is also stimulated via the Gβγ dimer [51]. Furthermore, the Gβγ subunit can also directly activate PLC ε [52].

PLC γ_1_ is localized in the cell cytosol and is regarded as the predominant cardiac PLC isozyme [53]. This isozyme is activated by GPCR and growth factor receptor tyrosine kinases, although a non-tyrosine kinase-mediated stimulation of PLC γ_1_ has also been observed [54]. Indeed, some of our work has suggested the existence of a tyrosine kinase and Gqα subunit cross-talk in adult rat cardiomyocytes [55]. In contrast, PLC δ_1_ is viewed to be the major PLC isozyme located at the sarcolemmal (SL) membrane. This has been attributed to the presence of basic amino acids in the N-terminal region of the pleckstrin homology domain of PLC δ_1_, which have a strong affinity for membrane PIP_2_ [56,57]. While intracellular Ca^2+^ levels have been shown to modulate PLC δ_1_ activity, a direct α_1_-AR induced activation of PLC δ isozyme by the dimeric G_h_ protein has also been reported [58,59].

## 3. Upregulation of PLC Isozymes in Pathological Cardiomyocyte Growth

### Transgenic and In Vivo Models of Cardiac Hypertrophy

The upregulation of PLC has been established in several diverse transgenic mice and other animal models of cardiac hypertrophy. In this regard, the activation of PLC-mediated signal transduction in cardiac hypertrophy in stroke-prone spontaneously hypertensive rats has been reported [60,61]. In volume overload-induced cardiac hypertrophy subsequent to an arteriovenous shunt, specific increases in PLC β_1_ and PLC γ_1_ isozyme gene, protein contents, and activities [62,63] have been observed. A similar activation of PLC β_3_ has been observed in pressure overload-induced cardiac hypertrophy due to aortic constriction in the rat [64]. In contrast, the activation of PKC isozymes has been observed in pressure overload cardiac hypertrophy in the guinea pig, subsequent to ligation of the descending thoracic artery, without any changes in PLC β_1_ and Gαq protein contents [65]. However, these investigators indicated that the stimulation of PKC isozymes could be a result of an increase in Gαq and PLC β_1_ activities instead of an upregulation of their expression [66]; it should be mentioned that PLC β_1_ activity was not measured in this study. Since mechanical stretch is an initiating feature for hypertrophy in response to hemodynamic overload, and that an increase in both Gqα and PLC β_1_ activities has been reported in stretched cardiomyocytes [29,30] as well as increased sympathetic nervous activity [67] in pressure-overload hypertrophy, it is therefore highly conceivable that stimulation of the α_1_-ARs increases PLC β isozyme activity in mechanical stress.

The stimulation of receptor-coupled Gqα in transgenic mouse models overexpressing Gqα results in cardiac hypertrophy [68,69,70,71], which potentially may be attributed to increases in PLC activity. However, the activation of PLC was found not to be associated with cardiac hypertrophy in two other different transgenic mouse lines expressing activated Gqα [72,73]. Although signal transduction mediated by specific PLC isozymes is an important component of the cardiomyocyte hypertrophic response, the abolition of signal transduction in PLC ε (−/−) mice results in cardiac hypertrophy that has been attributed to an increase in sensitivity to isoproterenol [66,74]. While the individual role of the different PLC isozymes in pathological cardiomyocyte growth still remains to be fully understood, from the information provided, it is apparent that PLC is an important contributor to the signaling processes leading to cardiac hypertrophy.

## 4. Isolated Cardiomyocytes for Assessing the Hypertrophic Response

The generation of IP_3_ in neonatal cardiomyocytes has been observed in response to norepinephrine and is considered to be attributed to the α_1_-AR-mediated activation of PLC β_1_ [75]. There are two splice variants of PLC β_1_ in the heart, PLC β_1a_ and PLC β_1b_, which differ in their C-terminal amino acid sequences [76]. While PLC β_1a_ is located in the cytosol, PLC β_1b_ is localized in the SL membrane, particularly in regions rich in caveolae (membrane lipid rafts), and where the α_1_-ARs are also localized [76]. Therefore, α_1_-AR-mediated signal transduction in response to norepinephrine can be considered to be due to PLC β_1b_. As a consequence, targeting of PLC β_1b_ is considered to have the potential to attenuate the development and progression of cardiac hypertrophy [76]. Indeed, an increase in cardiomyocyte growth with a concomitant increase in the ratio of protein to DNA as well as an increase in ANF levels have been reported in neonatal cardiomyocytes overexpressing PLC β_1b_ [77]. These observations demonstrated the involvement of α_1_-AR-PLC β_1b_ in the hypertrophic response and further strengthened the viability of PLC β_1b_ as a target for prevention/restriction of cardiac hypertrophy [77]. As already mentioned, PLC β_4_ is highly expressed in human left ventricular tissue and in view of its reported increase in mouse HL-1 cardiomyocytes exposed to different hypertrophic agents, it is conceivable that PLC β_4_ may play a complementary role in the signal transduction processes for pathological cardiomyocyte growth [49].

Although there is a large body of evidence on the involvement of PLC β isozymes in cardiac hypertrophy, the contribution of other myocardial PLC isozymes to the cardiomyocyte hypertrophic response is less defined, but information is now emerging. In this regard, depletion of PLC ε with siRNA in neonatal cardiomyocytes has been observed to reduce the cardiomyocyte response to a variety of hypertrophic agents [74]. However, even though a reduction in PLC ε was shown not to attenuate IP_3_ production, it was proposed that regionalized PLC activity was essential for this response.

Our attention has focused on determining the contribution of the different PLC isozymes in cardiac hypertrophy. Although the involvement of the α_1_-AR- PLC signaling axis has been confirmed by antagonism of the α_1_-AR with prazosin, as well as inhibition of PLC activity with a compound, U73122, subsequent studies have been conducted to dissect the signal transduction events and transcriptional parameters that influence the expression levels of the PLC genes [78,79,80]. The data presented in Table 1 show specific increases in the transcription factors, c-Fos and c-Jun in adult rat cardiomyocytes treated with norepinephrine and with phenylephrine; no changes in the other transcription factors (NFAT3, NFκB, MEF2C, and MEF2D) were observed [78]. These findings were suggestive of a specific and early upregulation of both c-Fos and c-Jun due to stimulation of the α_1_-AR under our experimental conditions.

Cardiomyocytes were treated with 5 µM norepinephrine (NE) or 1 µM phenylephrine (PhE) for 2 h. Transcription factor mRNA levels were measured by semi-quantitative RT-PCR and the data are presented as a percentage of the control values. These values are means ± S.E. of 5 experiments conducted with 5 different cardiomyocyte preparations. 

Subsequent studies demonstrated that both c-Fos and c-Jun have a regulatory role in the expression of PLC isozymes [79]. In this regard, by employing gene silencing techniques, it was found that transfection of cardiomyocytes with c-Fos siRNA prevented the norepinephrine-induced increases in PLC β_1_ and PLC β_3_ mRNA levels, but did not affect the norepinephrine-induced increases in PLC γ_1_ and PLC δ_1_ gene expression (Table 2A). In addition, silencing of c-Jun with siRNA not only inhibited the norepinephrine-induced increases in PLC β_1_ and PLC β_3_ mRNA levels but also prevented the increase in PLC δ_1_ mRNA levels in response to norepinephrine. Similarly, silencing of c-Jun did not attenuate PLC γ1 gene expression in response to norepinephrine (Table 2A). Furthermore, knockdown of both c-Fos and c-Jun inhibited the norepinephrine-induced activation of PLC isozymes as determined by the formation of inositol phosphates (Table 2B). These data demonstrated differential transcriptional regulation of PLC isozymes [79]. 

Since stimulation of the α_1_-AR was seen to result in a specific increase in the mRNA levels of both c-Fos and c-Jun, it is likely that α_1_-AR-PLC signal transduction is implicated in increases in c-Fos and c-Jun mRNA levels due to norepinephrine. It can be observed from Table 3 that both prazosin and U73122 blocked the increases in c-Fos and c-Jun mRNA levels in cardiomyocytes exposed to norepinephrine [78,79]. To further verify the participation of PLC in modulating transcription factor expression levels, cardiomyocytes were transfected with PLC isozyme-specific siRNA.

Cardiomyocytes were transfected with or without 5 nM siRNA and treated with NE (5 μM) for 2 h. Cardiomyocytes without any treatment served as control. PLC mRNA levels (A) were determined by semi-quantitative RT-PCR and data are presented as a percentage of the control value. PLC isozymes activities (B) were determined by measuring the hydrolysis of [^3^H]-PIP_2_ and are expressed as pmol/min/mg protein of inositol phosphates formed. These values are means ± S.E. of 5 experiments conducted with 5 different cardiomyocyte preparations. 

Cardiomyocytes were exposed to NE (5 μM) without and with prazosin (2 μM), U73122 (1 nM), or after transfection with 5 nM PLC isozyme siRNA for 2 h. c-Fos and c-Jun mRNA levels were determined by semi-quantitative RT-PCR and data are presented as a percentage of the control value. These values are means ± S.E. of 5 experiments conducted with 5 different cardiomyocyte preparations.

With the exception of silencing of PLC γ_1_, knockdown of PLC β_1_, β_3_, and δ_1_ genes was observed to prevent the increases in the mRNA levels of c-Fos and c-Jun (Table 3). These data indicated that the norepinephrine- α_1_-AR-mediated increases in c-Fos and c-Jun mRNA may involve signal transduction via some specific PLC isozymes.

The signaling events that regulate the expression levels of PLC isozyme genes were delineated through pharmacological and gene silencing interventions [79,80]. While prazosin and U73122 attenuated the increases in PLC isozyme gene expression in response to norepinephrine, silencing of the PLC gene with siRNA also prevented the norepinephrine-induced increases in PLC gene expression. In view of these observations, it was hypothesized that PLC activity can increase its own gene expression in response to stimulation with norepinephrine in adult cardiomyocytes (Table 4). Furthermore, it was demonstrated that PLC isozyme self-regulation of gene expression, may involve downstream PKC- and ERK1/2- signaling processes (Table 5 [80]).

Cardiomyocytes were exposed to NE (5 μM) without and with prazosin (2 μM), U73122 (1 nM), or after transfection with 5 nM PLC isozyme siRNA for 2 h. PLC isozyme mRNA levels were determined by semi-quantitative RT-PCR and data are presented as a percentage of the control value. These values are means ± S.E. of 5–10 experiments conducted with 5–10 different cardiomyocyte preparations.

Cardiomyocytes were exposed to different concentrations of PMA (0.1, 1.0, and 10.0 μM) and to NE (5 μM) without and with varying concentrations of Bis-1 (50, 100, and 200 nM) and PD98059 (2, 10, and 25 nM) for 2 h. PLC mRNA levels were determined by semi-quantitative RT-PCR and data are presented as a percentage of the control value. These values are means ± S.E. of 5 experiments conducted with 5 different cardiomyocyte preparations.

**Figure 2:**
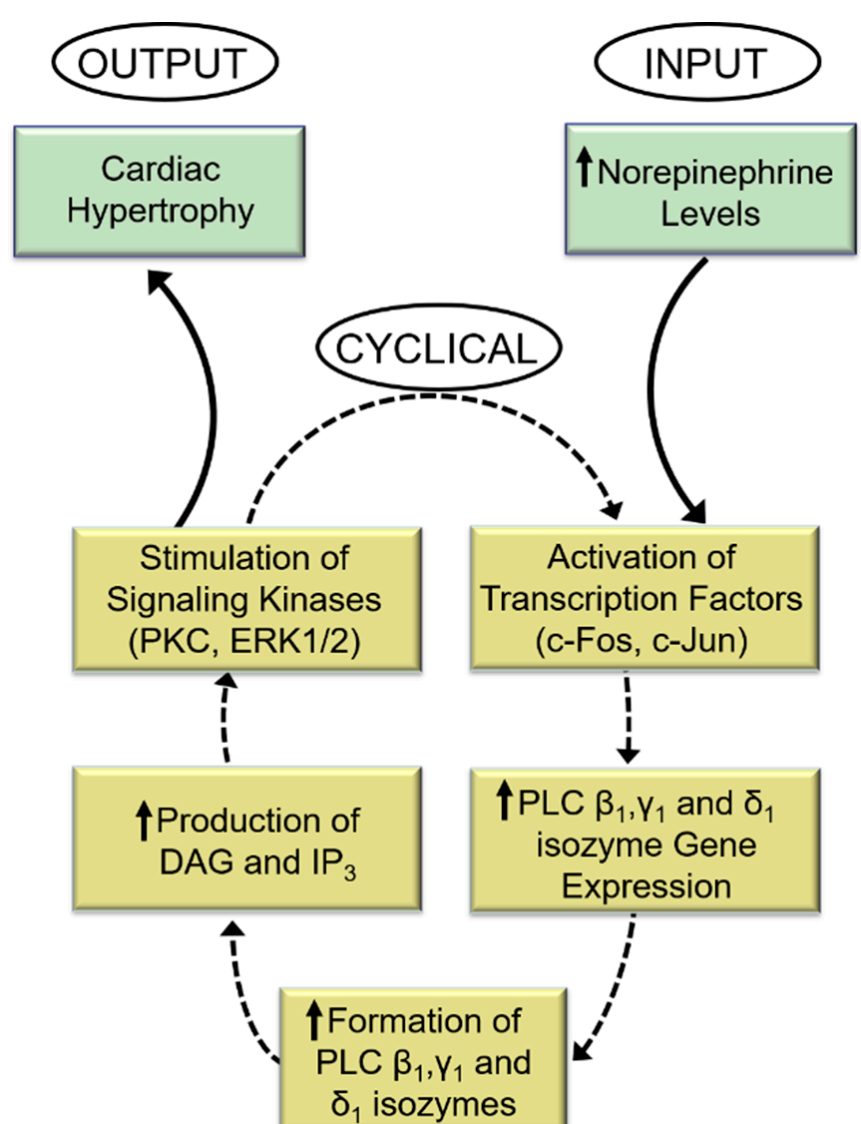
Role of phospholipase C in the perpetuation of cardiomyocyte growth response to norepinephrine. DAG = *sn*-1,2-diacylglycerol; IP_3_, inositol-1,4,5-trisphosphate. The response to increased levels of norepinephrine (input) is a cyclical process that produces an increase in the expression of PLC isozyme genes and subsequent higher generation of DAG and IP_3_ and stimulation of the signal for cardiac hypertrophy (output). The activation and amplification of PLC isozymes ensure continuation of this cycle of events for continuation of abnormal hypertrophic growth due sustained exposure to high levels of norepinephrine.

Indeed, while the PKC activator (phorbol myristate acetate, PMA) increased PLC gene expression, the PKC activity inhibitor (bisindolylmaleimide, Bis-1) markedly attenuated the norepinephrine-induced increases in PLC isozyme mRNA level. Similarly, blockade of ERK1/2 with PD98059 abolished PLC gene expression in response to norepinephrine. From the aforementioned, it is proposed that PLC activation is an early response to α_1_-AR activation by norepinephrine and that subsequent signal transduction events that augment PLC gene expression and activities may constitute a sequence of cyclical events designed to perpetuate cardiac hypertrophy and facilitate its ultimate transition into heart failure (Figure 2).

## 5. Evidence for Regression of Abnormal Cardiomyocyte Growth by α_1_-AR Blockade

From the above-mentioned discussion, it is evident that the α_1_-AR-Gqα-PLC signal transduction pathway has an important contribution to the hypertrophic response to norepinephrine. Accordingly, there are some experimental studies that have demonstrated that blockade of the α_1_-AR with prazosin mitigates the transition of cardiac hypertrophy to heart failure [81,82,83,84,85].

In addition, we have observed prazosin and metoprolol (a β_1_-AR receptor blocker) to reverse cardiac remodeling in the failing rat heart [86,87]. Furthermore, labetalol, a non-selective β-AR blocker, has been reported to reverse cardiac hypertrophy [88,89].

Importantly, several clinical investigations have revealed the advantageous effects of α_1_-AR antagonists including prazosin in failing hearts due to different etiologies [90,91,92,93]. It should be mentioned that while co-administration of prazosin and metoprolol in heart failure was observed not to exert any additive effects [94], the results of the COMET trial (Carvedilol or Metoprolol European Trial), revealed a greater benefit of carvedilol in heart failure than with metoprolol alone [95]. Taken together, these lines of evidence suggest that agents that exhibit a capability to block both α- and β-ARs can provide an improved outcome by attenuation of cardiac hypertrophy. However, it should be noted that the use of α_1_-AR blockers in patients with heart failure has been reported to produce no improvement in the condition [96,97]. Nonetheless, signal transduction mediated by the α_1_-AR-PLC pathway can be considered to play an essential contributory role in pathological cardiomyocyte growth. Furthermore, it can be proposed that the activation of this signaling pathway perpetuates cardiac hypertrophy that eventually progresses to heart failure. Although the literature has focused largely on antagonism the role of the It should be mentioned that in cardiomyocytes isolated from spontaneously hypertensive rats, α_2_-AR signaling is markedly attenuated [98]. Moreover, it has been suggested that α_2_-AR-mediated signal transduction counterbalances PLC-mediated signaling [99] indicating that cardiac SL α_2_-ARs may also be an important target [100] and potentially for the mitigation of cardiac hypertrophy.

## 6. Conclusions

We have extensively reviewed the literature regarding the involvement of phospholipid-mediated signal transduction mechanisms in different myocardial diseases [101,102,103,104,105,106], and recently the involvement of PLC in the cardiomyocyte hypertrophic response to norepinephrine [107] as well as the role of PLC in the catecholamine-induced increase in cardiomyocyte protein synthesis [108] has also been reviewed. In the present article, the activation of PLC and the regulation of its gene expression in cardiomyocyte hypertrophic response have been addressed. From the evidence provided, it can be suggested that the activation of specific PLC isozymes by norepinephrine is an important aspect of the signal transduction cascade that stimulates abnormal cardiomyocyte growth and that this pathway may constitute a sequence of cyclical actions that allow for the continuation of cardiac hypertrophy (Figure 2). There are several protein kinases that are activated by the PLC pathway, these include PKC, ERK1/2, and Ca^2+^/calmodulin-dependent kinase. These kinases have an important role as they, in turn, phosphorylate and activate some transcription factors including c-Fos and c-Jun that leads to PLC gene expression.

The reciprocal relationship between PLC activities and PLC gene expression is a characteristic feature that augments cardiac hypertrophy. It should be noted that it is not the intention of this review to exclude the role of the β_1_-AR Gs-protein-adenylyl cyclase system, which is largely responsible for initiating the development of cardiac hypertrophy. However, it is our contention that signal transduction through the α_1_-AR-Gqα-PLC axis plays a critical and complementary role in the initial phase of abnormal cardiomyocyte growth, and that signal transduction through this pathway is more significant in the late stage of cardiac hypertrophy as the β_1_-AR is downregulated at this phase. It should also be mentioned that while the focus of this review has been on the α_1_-AR-Gqα-PLC, there are other GPCRs that mediate the hypertrophic response. In this regard, the angiotensin II receptor blocker losartan has been observed to diminish PLC gene expression with a concomitant regression of cardiac hypertrophy [62]. Accordingly, it can be suggested that PLC has the potential to be viewed as an additional target for limiting pathological cardiomyocyte growth. 

## Figures and Tables

**Table 1:** Specific alterations in transcription factor gene expression in cardiomyocytes treated with norepinephrine and phenylephrine.

Transcription Factor mRNA Level (% of Control)						
	NFAT3 (99 bp)	NFκB (124 bp)	MEF2C (92 bp)	MEF2D (105 bp)	c-Fos (74 bp)	c-Jun (163 bp)
**Agonist**						
NE	110 ± 8	112 ± 9	121 ± 11	113 ± 6	268 ± 12 *	217 ± 6 *
PhE	125 ± 11	109 ± 8	127 ± 8	130 ± 13	261 ± 7 *	225 ± 8 *

* Significantly different (*p* < 0.05) vs. control value. Information presented is based on the data in our paper [78].

**Table 2:** Inhibition of norepinephrine the increases in PLC isozyme gene expression (A) and activities (B) in response to norepinephrine in cardiomyocytes transfected with c-Fos and c-Jun siRNA.

**A:**	**PLC mRNA Levels (% of Control)**			
	**β_1_ (114 bp)**	**β_3_ (230 bp)**	**γ_1_ (123 bp)**	**δ_1_ (190 bp)**
**NE**	219 ± 11 *	182 ± 18 *	168 ± 18 *	221 ± 18 *
**NE + cFos siRNA**	80 ± 21 ^#^	89 ± 11 ^#^	160 ± 15 ^#^	218 ± 17 ^#^
**NE + cJun siRNA**	75 ± 19 ^#^	91 ± 9 ^#^	170 ± 14 ^#^	74 ± 8 ^#^
**B:**	**Inositol Phosphates (pmol/min/mg Protein)**			
	**PLC β_1_**	**PLC β_3_**	**PLC δ_1_**	
**Control**	2.8 ± 0.6	4.1 ± 0.8	10.0 ± 1.4	
**NE**	6.8 ± 1.0 *	7.0 ± 1.3 *	17.1 ± 3.0 *	
**NE + cFos siRNA**	3.5 ± 0.7 ^#^	4.5 ± 0.8 ^#^	16.6 ± 2.4	
**NE + cJun siRNA**	3.0 ± 0.8 ^#^	3.9 ± 0.9 ^#^	11.8 ± 2.6 ^#^	

* Significantly different (*p* < 0.05) vs. control; ^#^ significantly different (*p* < 0.05) vs. NE. NE, norepinephrine; siRNA = small interfering RNA. Information presented is based on the data in our paper [79].

**Table 3:** Pharmacological and gene silencing interventions for the inhibition of increases in c-Fos and c-Jun gene expression levels in response to norepinephrine.

Condition	c-Fos mRNA Expression Levels	c-Jun mRNA Expression Levels
**NE**	214 ± 23 *	198 ± 20 *
**+Prazosin**	114 ± 11^#^	100 ± 8 ^#^
**+U73122**	83 ± 8 ^#^	95 ± 9 ^#^
**+PLC β_1_ siRNA**	110 ± 8 ^#^	85 ± 8 ^#^
**+PLC β_3_ siRNA**	105 ± 7 ^#^	95 ± 11 ^#^
**+PLC γ_1_ siRNA**	175 ± 25 *	180 ± 13 *
**+PLC δ_1_ siRNA**	108 ± 7 ^#^	80 ± 7 ^#^

* Significantly different (*p* < 0.05) vs. control. ^#^ significantly different (*p* < 0.05) vs. NE. NE, norepinephrine; siRNA = small interfering RNA. Information presented is based on the data in our papers [78,79].

**Table 4:** Attenuation of the increases in phospholipase C gene expression due to norepinephrine by pharmacological and gene silencing interventions.

	PLC Isozyme mRNA Levels (% of Control)			
Condition	β_1_	β_3_	γ_1_	δ_1_
**NE**	201 ± 9 *	188 ± 8 *	181 ± 9 *	159 ± 8 *
**+Prazosin**	99 ± 11 ^#^	102 ± 4 ^#^	120 ± 5 ^#^	90 ± 4 ^#^
**+U73122**	68 ± 5 ^#^	80 ± 4 ^#^	103 ± 11 ^#^	67 ± 12 ^#^
**+PLC β_1_ siRNA**	90 ± 8 ^#^	-	-	-
**+PLC β_3_ siRNA**	-	80 ± 9 ^#^	-	-
**+PLC γ_1_ siRNA**	-	-	61 ± 7 *	-
**+PLC δ_1_ siRNA**	-	-	-	60 ± 8 ^#^

* Significantly different (*p* < 0.05) vs. control. ^#^ significantly different (*p* < 0.05) vs. NE. NE, norepinephrine; siRNA = small interfering RNA. Information presented is based on the data in our papers [79,80].

**Table 5:** PLC isozyme gene expression in adult rat cardiomyocytes treated with phorbol 12-myristate 13-acetate, bisindolylmaleimide, or PD98509.

	PLC mRNA Levels (% of Control)			
Treatment	β_1_	β_3_	γ_1_	δ_1_
**PMA (µM)**				
**10.1**	118 ± 11	131 ± 8	110 ± 10	108 ± 10
**1.0**	190 ± 12 *	183 ± 9 *	176 ± 13 *	161 ± 15 *
**10.0**	175 ± 11 *	171 ± 10 *	130 ± 12 *	123 ± 12 *
**NE (µM)**				
**5.0**	223 ± 11 *	192 ± 19 *	186 ± 15 *	193 ± 15 *
**+Bis-1 (nM)**				
**50**	95 ± 10 ^#^	96 ± 9 ^#^	111 ± 14 ^#^	100 ± 8 ^#^
**100**	83 ± 8 ^#^	80 ± 12 ^#^	100 ± 10 ^#^	82 ± 9 ^#^
**200**	82 ± 9 ^#^	75 ± 6 ^#^	82 ± 9 ^#^	77 ± 9 ^#^
**+PD98059 (nM)**				
**2**	107 ± 11 ^#^	90 ± 9 ^#^	117 ± 14 ^#^	120 ± 11 ^#^
**10**	84 ± 9 ^#^	87 ± 11 ^#^	103 ± 9 ^#^	94 ± 8 ^#^
**25**	77 ± 8 ^#^	82 ± 7 ^#^	82 ± 11 ^#^	90 ± 12 ^#^

* Significantly different (*p* < 0.05) vs. control; ^#^ significantly different (*p* < 0.05) vs. NE. NE, norepinephrine; PMA, phorbol myristate acetate; Bis-1, bisindolylmaleimide Information presented is based on the data in our paper [80].

## Data Availability

Not applicable.
